# Using hierarchical clustering methods to classify motor activities of COPD patients from wearable sensor data

**DOI:** 10.1186/1743-0003-2-16

**Published:** 2005-06-29

**Authors:** Delsey M Sherrill, Marilyn L Moy, John J Reilly, Paolo Bonato

**Affiliations:** 1 Dept of Physical Medicine and Rehabilitation, Harvard Medical School, Spaulding Rehabilitation Hospital, Boston MA, USA; 2 Dept of Medicine, Harvard Medical School, Brigham and Women's Hospital, Boston MA, USA; 3 The Harvard-MIT Division of Health Sciences and Technology, Cambridge MA, USA

## Abstract

**Background:**

Advances in miniature sensor technology have led to the development of wearable systems that allow one to monitor motor activities in the field. A variety of classifiers have been proposed in the past, but little has been done toward developing systematic approaches to assess the feasibility of discriminating the motor tasks of interest and to guide the choice of the classifier architecture.

**Methods:**

A technique is introduced to address this problem according to a hierarchical framework and its use is demonstrated for the application of detecting motor activities in patients with chronic obstructive pulmonary disease (COPD) undergoing pulmonary rehabilitation. Accelerometers were used to collect data for 10 different classes of activity. Features were extracted to capture essential properties of the data set and reduce the dimensionality of the problem at hand. Cluster measures were utilized to find natural groupings in the data set and then construct a hierarchy of the relationships between clusters to guide the process of merging clusters that are too similar to distinguish reliably. It provides a means to assess whether the benefits of merging for performance of a classifier outweigh the loss of resolution incurred through merging.

**Results:**

Analysis of the COPD data set demonstrated that motor tasks related to ambulation can be reliably discriminated from tasks performed in a seated position with the legs in motion or stationary using two features derived from one accelerometer. Classifying motor tasks within the category of activities related to ambulation requires more advanced techniques. While in certain cases all the tasks could be accurately classified, in others merging clusters associated with different motor tasks was necessary. When merging clusters, it was found that the proposed method could lead to more than 12% improvement in classifier accuracy while retaining resolution of 4 tasks.

**Conclusion:**

Hierarchical clustering methods are relevant to developing classifiers of motor activities from data recorded using wearable systems. They allow users to assess feasibility of a classification problem and choose architectures that maximize accuracy. By relying on this approach, the clinical importance of discriminating motor tasks can be easily taken into consideration while designing the classifier.

## Background

### Field Monitoring of Motor Activities

During the past decade, the interest of researchers and clinicians has focused on wearable sensors and systems as means to monitor motor activities in the home and the community settings [1-3]. Objective measures of physical activities outside of the clinical setting are sought because subject report is notoriously inaccurate. For instance, Pitta et al. [4] showed that subjects overestimated time spent walking, cycling, and standing, and underestimated time spent sitting and lying. They used a triaxial accelerometer to quantify time spent in a standardized protocol of walking, cycling, standing, sitting, and lying in patients with chronic obstructive pulmonary disease (COPD). They videotaped the performance of the protocol and asked subjects to estimate time spent in each activity. Differences between outcomes from videotape and the accelerometer ranged from 0% (sitting) to 10% (lying). In contrast, differences between videotape and patient report ranged from 18% (lying) to 59% (walking).

The simplest device to monitor motor activities consists of a single accelerometer positioned on the body segment mostly involved in the motor activity of interest [3]. Pedometers and step counters are the most popular among these devices. Since the mid-nineties, researchers have utilized this approach to estimate overall level of activity and energy expenditure (e.g. [5,6]). A number of studies have been devoted to investigate clinical uses of systems based on a single accelerometer. Among others, Steele et al. [7,8] measured human movement in three dimensions over 3 days and showed that the magnitude of the acceleration vector is correlated with existing clinical measures such as the six-minute walk distance, FEV1 (forced expiratory volume in 1s), dyspnea, and Physical Function domain of health-related quality of life. Moy et al [9] showed that monitoring of ambulation in patients with COPD over two-week periods in the home environment correlates with global assessments of health-related quality of life such as General Health and Mental Health on the SF-36. The limitations of these devices are that they record only ambulation, do not assess upper arm movements, cannot discriminate changes in grade and intensity of workload, and do not assess concomitant systemic responses.

To overcome at least some of the limitations of devices based on a single accelerometer, researchers have developed ambulatory and wearable systems to simultaneously monitor the movement of multiple body segments. Although there is a trade-off in simplicity of use, the ability of these systems to measure the orientation (due to the effect of gravity) and acceleration of individual segments, as well as intersegmental coordination, has opened the door to a variety of applications requiring the identification of specific activities. In the late nineties, several research teams [10-13] attained greater than 70% sensitivity to each of 4 classes of activity: sitting, standing, lying, and dynamic movement. Researchers used data-loggers connected to miniature accelerometers that were attached to the sternum/waist (bi- or triaxial) and one or both thighs (uniaxial), and data were collected under controlled laboratory conditions. Using a 5-sensor configuration, Foerster and Fahrenberg [13] subdivided the 4 classes into 13 separate tasks: 3 types of sitting, 4 types of lying, 5 types of dynamic motion, and standing. Sensitivity for the different tasks ranged between 82 and 98%.

During the past five years, numerous research teams further developed the potential of accelerometer-based systems to monitor motor activities in the field. Among others, Schasfoort et al [14] first focused on quantifying upper body activity by means of accelerometers. The development of the technique was followed by its application to the assessment of the degree of impairment and activity limitation in patients with complex regional pain syndrome type I [15]. Sherrill et al [16] explored the use of an activity monitor to gather information related to the level of independence of individuals similar to what is typically accomplished by a Functional Independence Measure assessment [17]. Bussmann et al [18] utilized an accelerometer-based system to assess mobility in transtibial amputees. Other research teams explored the use of accelerometers to monitor motor patterns in patients with Parkinson's disease [19-22] and in post-stroke individuals following rehabilitation [23,24].

In the studies mentioned thus far, the algorithms developed and utilized to identify different motor activities constitute a key point of the proposed methods. Various approaches have been developed by our team and others ranging from the application of simple rule-based classifiers [12,23,25,26] to complex pattern recognition algorithms involving a combination of neural networks and neuro-fuzzy inference systems [16,19]. When clear differences are known *a priori *to exist among the motor activities to be identified (e.g. sitting vs. walking), simple rule-based classifiers are usually sufficient. However, when the activities of interest are complex, and the distinctions among them more subtle and subject to individual variability, more advanced pattern recognition algorithms are called for. In most real-world situations, the set of motor activities under investigation includes members of both categories. A hierarchical approach such as that proposed by Mathie et al. [2] appears to offer a suitable compromise. In Mathie et al.'s classification scheme, movements were categorized very generally at the top of the hierarchy (activity vs. rest) and then subdivided, over 4 additional levels, into progressively more specialized submovements using a binary decision at each node. The authors achieved an average 97% accuracy in identifying 15 submovements (7 static postures, 5 postural transitions, and 3 dynamic categories).

The methods described in this paper can be viewed as an extension of Mathie et al.'s framework to include a greater variety of dynamic activities of the upper and lower extremities. In particular, for the COPD population (the target patient population of the application described in this manuscript) it is important to distinguish subtypes of ambulation because they correspond to different levels of physical exertion: walking up stairs or up an incline is more fatiguing than walking on level ground or descending stairs or an incline. For such activities, it is not clear at the outset which features of the accelerometer data will best distinguish these conditions. Indeed, there is no guarantee that the data even contain sufficient information to make such distinctions in all cases, or in every subject, due to individual variations in body type and pattern of movement. Our essential approach is to rely on clustering techniques to explore the data set for each individual, assess whether distinct clusters correspond to different motor tasks, determine whether simple rules can contrast clusters associated with different tasks, and evaluate the need for merging clusters when the information derived from accelerometer data appears insufficient to sort out different motor tasks.

### Medical Application

To demonstrate the efficacy of the proposed approach, a data set recorded from patients with COPD is utilized. Monitoring motor activities in patients with COPD is of great clinical interest. COPD is predicted to be the third most frequent cause of death in the world by 2020 [27]. It afflicts more than 15 million Americans, results in more than 15 million physician office visits each year, and causes approximately 150 million days of disability per year [28]. The total direct cost of medical care related to COPD is approximately $15 billion per year [29]. COPD is a steadily progressive, debilitating disease for which existing medical therapies are largely ineffective. With decreasing lung function, patients are at increased risk for hospitalizations, need for supplemental oxygen therapy, decreased exercise capacity, and death. Physical exercise in particular is a crucial component to the medical treatment of COPD to prevent deconditioning, to improve health-related quality of life, and to optimize response to surgical interventions [30]. Hence the improvement of exercise capacity is a major goal in the treatment of patients with COPD.

Celli et al [31] showed that exercise tolerance, which reflects the systemic consequences of COPD, added to the predictive power to predict mortality of FEV1, the long-held 'gold standard' measure of disease progression in COPD. Exercise tolerance can be assessed in the clinical setting via the progressive incremental cardiopulmonary exercise test. Performed either on a treadmill or stationary bicycle, cardiopulmonary exercise testing yields integrative information about the metabolic, cardiovascular, and ventilatory processes that occur during exercise. Exercise tolerance can also be measured indirectly via timed walking tests, the advantages of which are simplicity, minimal resource requirements, and general applicability. However, the disadvantages of timed walking tests include dependence on patient and administrator motivation, effects of learning, and a potential for inter-test variability if the administrators give differing instructions or encouragement during separate tests over time [32].

Furthermore, neither the cardiopulmonary exercise test nor timed walking tests capture work performed by the upper extremities. It has been demonstrated that unsupported arm exercise in patients with COPD produces dyssynchronous breathing, and thus dyspnea and sensation of muscle fatigue [33]. During unsupported arm work, the accessory muscles of inspiration help position the torso and arms. It is hypothesized that the extra demand placed on these muscles during arm exertion leads to early fatigue, an increased load on the diaphragm, and dyssynchronous thoracoabdominal inspirations. Therefore accurate measurement of upper as well as lower extremity exercise capacity is important in assessing these patients.

Patients with COPD experience daily fluctuations in their clinical status, with "good and bad days" occurring as a function of airway secretions, humid weather, and other environmental factors. Moreover, COPD patients demonstrate widely variable exercise capacities even when they have identical degrees of airflow obstruction by pulmonary function tests [34]. These factors strongly motivate the development of a wearable, individually-customizable system to monitor activity in the home and community for days or weeks at a time as a supplement (or alternative) to controlled laboratory tests administered at a single point in time. To date, a number of researchers [7,8,26,30,35] have conducted preliminary studies to evaluate the relevance of field measures in COPD patients with encouraging results. It is thus particularly appropriate to utilize data recorded from COPD patients as a demonstration of the motor activity classification techniques proposed in this paper. In the following sections, we summarize the data collection protocol, describe the procedures to estimate features of the acceleration data, demonstrate the use of clustering methods for analysis of the feature sets, and discuss the generalization of the proposed approach to building classifiers of motor activities from field data.

## Methods

### Data Collection

**Figure 1:**
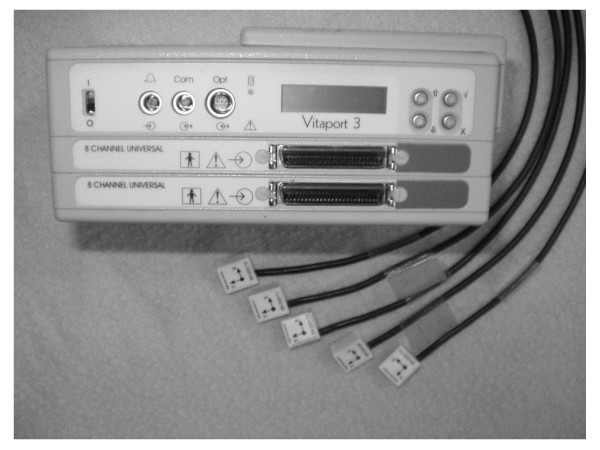
**Ambulatory recorder & accelerometers**. This system was utilized to gather accelerometer data from right and left forearm and right and left thigh from COPD patients performing a set of motor tasks in a controlled clinical environment. The sensor units shown in the picture are the biaxial accelerometers used in the study.

We gathered data from six individuals with severe COPD in a controlled clinical environment (Brigham & Women's Hospital Division of Pulmonary Rehabilitation). The subjects ranged in age from 51 to 80 years (mean age 63). Biaxial accelerometers were mounted on the lateral aspect of each subject's right and left forearm (approximately 10 cm proximal to the wrist joint) and on the lateral aspect of the right and left thigh (approximately 10 cm proximal to the knee joint). The sensitive axes were oriented to capture accelerations in the up-down and anteroposterior directions. Note that location of the sensors is described assuming a reference position of upright stance with arms at the sides and palms facing the midline of the body. An additional biaxial sensor was placed on the sternum to sense up-down and mediolateral motions of the trunk. Subjects were outfitted with a Vitaport 3 ambulatory recorder (Temec B.V., The Netherlands, shown in Figure 1), worn about the waist, to digitally sample (128 Hz) and store 10 channels of data continuously throughout the experiment. Care was taken to secure wires and minimize the impact of the system on the ability of patients to move freely.

The subjects were asked to perform 10 tasks according to a pre-defined protocol for at least one minute each. The protocol included three aerobic exercises typical of the prescribed pulmonary rehabilitation exercise regimen for these patients (walking on a treadmill, cycling on a stationary bike, and cycling on an arm ergometer), five tasks representing ambulation in a free-living environment (level walking in a hallway, ascending/descending a ramp, and ascending/descending stairs), and two other free-living activities, folding laundry in a seated position and sweeping the floor with a broom. These last two motor tasks were considered to assess whether it is possible to reject tasks that are somehow similar from a biomechanical point of view to the ones of interest, i.e. aerobic exercises and tasks representing ambulation. Identifying the full range of movement conditions would allow the assessment of patients' overall mobility in addition to their compliance with a prescribed exercise routine. Note that for certain tasks, such as climbing stairs, it was not possible to gather data continuously for an entire minute in every subject due to the physically demanding nature of those tasks. The experimenter kept a written log of the subject's activities and used a manual marker to segment the recording. The experimental protocol was reviewed and approved by the Brigham & Women's Hospital panel of the Partners HealthCare Human Research Committee.

**Figure 2:**
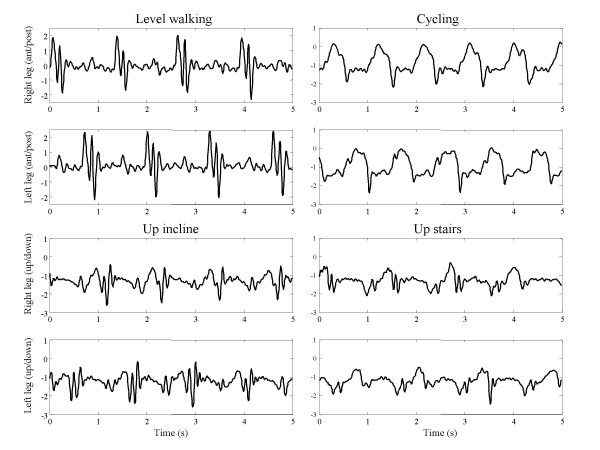
**Accelerometer data samples**. Accelerometer signals are shown over a window of 5s corresponding to a few cycles of the following motor tasks: level walking, cycling, walking up an incline, and walking up stairs. Data are shown for the accelerometers positioned on left and right thigh with axes oriented in the antero-posterior and up and down directions.

Examples of accelerometer signals for a few motor tasks from one subject are shown in Figure 2. Data are presented for four motor tasks, i.e. level walking in a hallway, cycling, ascending a ramp, and ascending stairs. Signals from the accelerometers positioned on the right and left legs oriented in the antero-posterior and up-down directions are plotted. These examples demonstrate differences and similarities in patterns of accelerometer data across motor tasks. For instance, data related to cycling are noticeably different from data related to level-walking. On the other hand, more subtle differences mark accelerometer data recorded while the subject was ascending stairs and signals gathered while the subject was ascending a ramp.

### Pre-processing and Feature Extraction

All processing routines were developed using Matlab (The MathWorks, Natick MA). Data were digitally filtered (5^th ^order elliptical lowpass, fc = 15 Hz, transition bandwidth 1 Hz, passband tolerance 0.5 dB, minimum stopband attenuation 20 dB, non-causal implementation) to remove high-frequency (noise) components unrelated to limb or trunk movement. Further, to separate components related to applied accelerations from those related to body segment orientation changes, a highpass digital filter was applied (2^nd ^order elliptical, fc = 0.5 Hz, transition bandwidth 0.5 Hz, passband tolerance 0.5 dB, minimum stopband attenuation 20 dB, non-causal implementation).

Extraction of epochs for further analysis was performed by sliding a 3s window through the recording at 1s intervals to extract the epochs. Note that this resulted in a 66% overlap between successive epochs. Then the following 9 features were extracted per epoch for each channel (or pair of channels, as indicated):

I. Time series features (3):

• Mean (prior to highpass filtering) was calculated as a measure of limb orientation and/or posture (all other features were derived from the highpass filtered data)

• RMS energy for each channel was calculated as a measure of magnitude of the overall acceleration applied to each body segment

• Range of each channel, a measure of peak acceleration

II. Spectral features (2):

• Dominant frequency component (i.e. 0.5 Hz bin with greatest energy) between 0.5 and 15 Hz

• Ratio of energy in dominant frequency component to the total energy below 15 Hz (an estimate of how much the signal is dominated by a particular frequency, i.e. its periodicity)

III. Correlation features (4):

• Range of autocorrelation function, a measure of the modulation of the signal (unbiased estimate)

• Value of the crosscorrelation function at zero lag (for all possible pairs of arm and leg channels), an approximate measure of intersegmental coordination.

• Peak value of the crosscorrelation function (for time-lags between -0.5 to 0.5 s), a measure of similarity of the movement patterns across body segments.

• Time-lag corresponding to the peak of the crosscorrelation function, which is a measure of the delay between movement of pairs of body segments

All features were assessed initially for consistency and variability across tasks using data visualization techniques. Certain features were excluded from further analysis of motor tasks associated with ambulation because they were found to interfere with reliable separation of these tasks. First, all features derived from sensors on the arms were excluded because their position can vary greatly. For instance, during a particular ambulatory task, the individual might swing his or her arms freely, hold on to a railing with one arm, or carry an object, whereas the goal is to identify the task regardless of such variations. Second, because the present aim is to identify the task regardless of speed, the dominant frequency feature for all channels was excluded because of its dependence on speed of locomotion. It is foreseeable that one could use this feature in the future in order to assess speed, which would be useful for marking conditions that are more physically taxing. In total there were 48 feature values per epoch in the ambulatory task analysis. Data were normalized across subjects. Principal components analysis [36] was performed to further reduce the dimensionality by transforming the data and retaining the first 6 components, which accounted for about 90% of the total variance. This step was necessary due to the small sample size.

### Analysis Procedures

The first stage in assessing the degree of similarity among classes was to visualize the reduced feature set in two dimensions with a scatter plot of the 1^st ^and 2^nd ^principal components. This was useful to build intuition about the structure of the data set, but a more objective method for similarity analysis is desirable from an automation standpoint. An objective measure of similarity would enable more systematic analysis of how task identification accuracy is affected by the merging of classes.

In order to measure the distinguishability of a subset of tasks on the basis of features derived from accelerometer data, clusters were defined based on class labels, and then the correspondence between labels and the natural groupings in the data was measured. Because we start with knowledge of the data labels, this is a reversal of the classic unsupervised learning paradigm where clusters are defined based on properties of the data and then used to label the data. In the unsupervised problem, the number of clusters is rarely known a priori. A typical approach is to try a range of possible values for the number of clusters, and then choose the clustering that maximizes a pre-defined cluster quality index (CQI). Our approach uses CQI to measure cluster similarity by calculating its value for each pair of clusters.

Two of the most widely cited CQIs in the machine learning literature are Dunn's index [37] and the Davies-Bouldin index [38]. Bezdek and Pal [39] presented a framework for generalizing Dunn's index so that virtually any combination of metrics for cluster separation and cluster size could be used to define an index of cluster quality. The Generalized Dunn's "intercluster distance" V_GD _for a given cluster pair is the separation between clusters normalized by their average diameter (hence favoring tight, spherical groupings spaced far apart):



The separation, δ, and diameter, Δ, can be computed in a variety of ways. Bezdek and Pal [39] presented six possible methods for computing δ and three methods for computing Δ, and evaluated the performance of all possible combinations on six benchmark data sets. Based on the successful performance results obtained in their simulations, we selected the following definitions of δ and Δ:





In Eq. 1–3, X_i _denotes the set of data points in the cluster corresponding to the i^th ^task, x_i _denotes a data point contained in X_i _(i.e. a vector of feature values derived from one epoch of sensor data), |X_i_| the number of data points in the i^th ^cluster, and μ_i _the centroid of X_i _(i.e. mean over all x_i _in X_i_). All vector distances are Euclidean, i.e. . The separation δ is the sum of the pairwise Euclidean distances between the centroid of one cluster and all points in the other cluster, and vice versa, divided by the total number of points in both clusters. Cluster diameter Δ is the average distance between data points in the cluster and the cluster centroid, multiplied by a factor of 2 to convert each radius to a diameter.

Having chosen a CQI to measure similarity, the next step was to define a hierarchy based on this information. Specifically, we used a linkage algorithm to build a dendrogram, a diagram in which similar objects are joined by links whose vertical position indicates the level of similarity between the objects. The average linkage algorithm, or UPGMA (Unweighted Pair Group Method with Arithmetic Averages [40]), was selected because of its demonstrated robustness to outliers [39]. This algorithm forms links between two objects based on the average distance between all pairs of lower-ranking objects. From the dendrogram, a sequence of merging steps was derived starting from the bottom level (no merging), and moving up one node at a time, where each node represents the merging of two lower nodes.

### Implementation and Testing

To assess the effect of successive merges on the accuracy of ambulatory task discrimination, a simple classifier was applied at each point in the sequence. Linear discriminant analysis (LDA) was selected for classification because its parameterization is minimal and it is therefore well suited to small data sets. Each level of merging was trained and tested independently with a balanced set of data; i.e. a data set sampled equally from each class. 75% of samples in the data set were used to train the classifier, and the remaining 25% were used in the testing. In addition, the entire training and testing process was repeated for 100 rotations of the data set so that the performance estimates (sensitivity and misclassification) would be less dependent on epoch selection and less sensitive to outliers. *Sensitivity *was defined as the number of times a task was correctly detected divided by the number of epochs corresponding to that task. *Misclassification *was defined as the number of identifications of a particular task arising from other tasks (i.e. incorrect detections of that task) divided by the number of epochs corresponding to other tasks.

## Results

### High-level Classification

At the top level of the hierarchy, the set of 10 tasks was split into three subcategories (ambulatory, sedentary with legs moving, and sedentary with legs stationary) using a simple threshold-based approach similar to that of Mathie et al [2]. For all six subjects, 100% sensitivity and 0% misclassification were achieved by the following criteria:

1) If mean of right thigh accelerometer (up-down axis) is greater than 0.6 g, task is sedentary; otherwise, task is ambulatory.

2) If task is sedentary and RMS of right thigh accelerometer (anteroposterior axis) is high (e.g. greater than 0.1 g), legs are moving; otherwise, legs are stationary.

**Figure 3:**
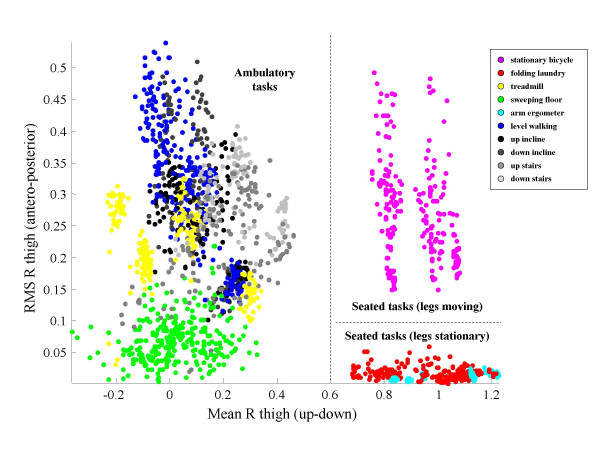
**High level separation of tasks (6 subjects)**. A scatter plot of the root mean square (RMS) value of the accelerometer data recorded in the antero-posterior direction from the right thigh vs. the mean value of the accelerometer data recorded from the same sensor unit in the up-down direction demonstrates that certain categories of tasks can be easily discriminated using a simple ruled-based approach. In fact, the plane can be divided into three regions containing the samples associated with motor tasks related to ambulation, motor tasks performed in a seated position with legs moving, and motor tasks performed in a seated position with legs stationary respectively.

In Figure 3, mean of the right thigh accelerometer (up-down axis) and RMS of the right thigh accelerometer (anteroposterior axis) are plotted for epochs representing all six subjects studied in order to demonstrate the efficacy of this approach. However, it is clear that more features will need to be taken into account in order to make further distinctions among tasks, and it is uncertain whether there is sufficient information in the data to make such distinctions in all cases. In the following we demonstrate the use of the CQI/cluster merging methods described earlier by focusing on identification of 6 ambulatory tasks: walking on a treadmill, level walking in a hallway, ascending/descending stairs, and ascending/descending a ramp.

### Ambulatory Task Classification

**Figure 4:**
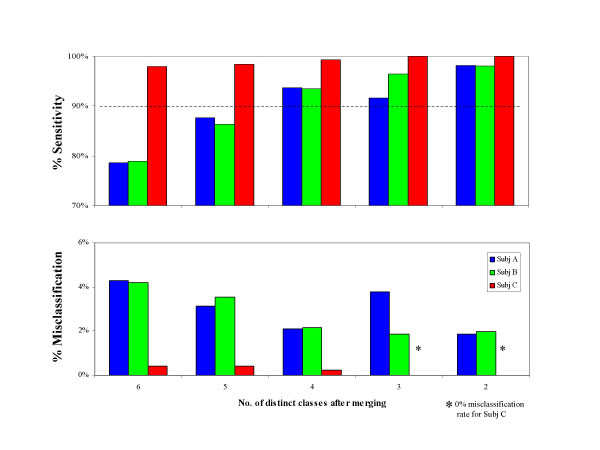
**Merging clusters for ambulatory tasks**. The barplots show sensitivity and misclassification for different levels of merging for the three subjects from which it was possible to gather sufficient data to explore discriminating among motor tasks associated with ambulation. While for Subj C an accurate discrimination of 6 tasks was obtained and thus no dramatic change is shown in sensitivity and misclassification when merging clusters, for Subj A and Subj B the increase in sensitivity and decrease in misclassification when merging clusters is significant. Sensitivity above 90% can be achieved while discriminating among 4 motor tasks.

Results for LDA-based classification of six ambulatory tasks are summarized in Figure 4. Only three out of the six subjects had at least 25 epochs available for all ambulatory tasks, therefore results are shown only for these three subjects (herein referred to as A, B, and C). For subjects A and B, sensitivity improved from 79% to 98% and misclassification decreased from 4.2% to 1.9% as the number of clusters decreased. For subject C, the merging of tasks did not lead to substantial improvement because accuracy was already quite high in the unmerged case.

Overall it appears that the method strongly favors merging tasks as much as possible. This is not a surprising result since the probability of correctly classifying a sample by chance increases from 17% to 50% as the number of clusters decreases from 6 to 2. The final decision about what level of merging is appropriate must take into consideration the context of the application. For instance, in the case of COPD activity monitoring, the eventual goal is to track physiological response over time associated with variously strenuous activities. Therefore we would hesitate to merge the tasks, for instance, of stair ascent and stair descent because of the very different metabolic costs associated with those activities. However, merging level walking together with walking down an incline is an acceptable loss of refinement if the overall detection accuracy is improved. Alternatively, an application might call for a minimum level of sensitivity, in which case one would choose the minimally merged set (i.e. that with the greatest number of distinct clusters) meeting that criterion. For example, setting a minimum sensitivity of 90% would lead to selection of the 4-cluster configuration for subjects A and B and selection of the original unmerged configuration for Subject C.

**Figure 5:**
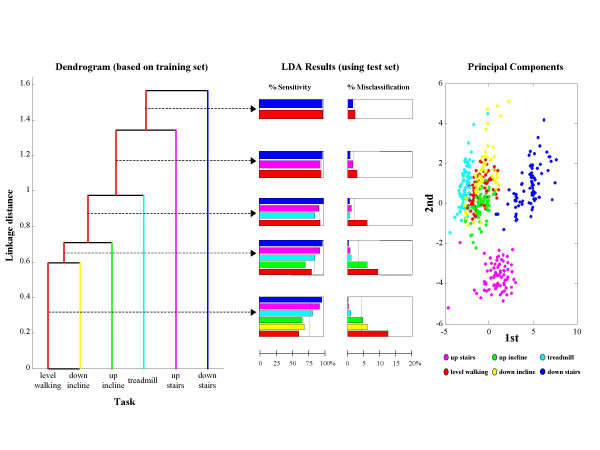
**Classifier results for Subj B**. Dendrogram, results of the LDA, and scatter plot of 1^st ^and 2^nd ^principal components are shown for Subj B. The scatter plot shows that while the clusters associated with walking up stairs and walking down stairs are clearly separated, the clusters associated with the other motor tasks significantly overlap. This is consistently shown, but in a more quantitative way, by the dendrogram that also suggests a strategy for merging clusters. When such strategy is adopted and an LDA algorithm is used, sensitivity and misclassification improve as shown by barplots. Dotted lines in the barplots are indicative of the mean value of sensitivity and misclassification across tasks.

**Figure 6:**
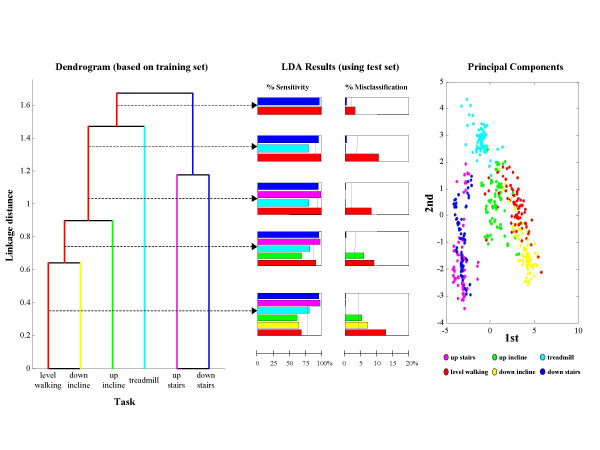
**Classifier results for Subj A**. Dendrogram, results of the LDA, and scatter plot of 1^st ^and 2^nd ^principal components are shown for Subj A. The information is herein presented as in Figure 5. However, different relationships among clusters are shown in this figure. Accordingly, a different strategy to merge clusters was adopted.

**Figure 7:**
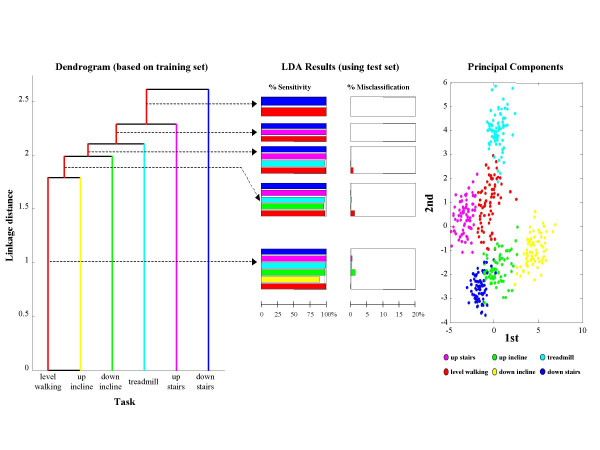
**Classifier results for Subj C**. Dendrogram, results of the LDA, and scatter plot of 1^st ^and 2^nd ^principal components are shown for Subj C. Contrary to what seen for Subj A and B, for Subj C the overlap among clusters is minimal and thus merging clusters does not appear to be necessary.

Detailed results for these subjects are shown in Figures 5, 6, and 7. Dendrograms of the cluster hierarchy, bar plots of percent sensitivity and misclassification by task, and scatter plots of the 1^st ^and 2^nd ^principal components of the unmerged configuration are shown for comparison. All three plots within a figure share a common color scheme.

For subject B, Figure 5 illustrates how the cluster hierarchy shown in the dendrogram at left reflects the internal structure of the data that is visualized in the scatter plot at right. Specifically, the bottom three tasks in the dendrogram (level walking, down incline, and up incline), with a fairly low linkage distance (0.6–0.7) are those with the most overlap in the scatter plot. The next level up in the dendrogram is walking on a treadmill, and in the scatter plot it is apparent that the corresponding points form a cluster that is near but not overlapping with the first three. The remaining two tasks are well separated in the scatter plot from the first four tasks and from one another, and in fact the linkage distances for these tasks are relatively high (1.4–1.6).

In Figure 6, the dendrogram for subject A is also reflective of the relationships among task clusters that are evident in the accompanying scatter plot. Namely, there is a clear three-way split in the scatter plot data, which corresponds to three natural groupings. What is suggested by the scatter plot corresponds to the second and third levels of merging in the dendrogram, with the task of walking on a treadmill showing the greatest linkage distance from the other two sections. This example also shows that the dendrogram is not strictly left-branching in all cases.

In the scatter plot for Subject C, shown at right in Figure 7, all six of the ambulatory tasks are well separated on the basis of just the first two principal components. Indeed, every task is identified with high accuracy, as seen in the bar plots for the performance characteristics. The structure of the dendrogram is consistent with these results as well, because even the lowest tier has a comparatively high linkage distance (≥ 1.75). This example demonstrates that merging is not necessary in every case.

## Discussion

We began this paper by reviewing recent work on using accelerometers to monitor motor activities in the laboratory and field. In particular, we focused on Mathie et al's [2] hierarchical framework as a useful way to formulate the problem, and developed a methodology that would extend this framework to handle more complex dynamic tasks involving the upper and lower extremities. The approach we have described combines existing cluster analysis techniques (i.e. CQI, average linkage, dendrograms) in a way that is, to our knowledge, novel. To demonstrate the application of this approach to a real data set, the application of monitoring exercise and free-living activities in subjects with COPD by means of accelerometers was used. High-level classifications of the COPD data did not require use of special techniques. Separation of tasks into three primary groups was easily accomplished using thresholds on two of the features derived from the accelerometer signals for data across all 6 subjects. However for the discrimination of ambulatory tasks the merging technique was necessary in two out of the three subjects for which enough data were available to explore the classification of ambulatory tasks. Merging tasks was necessary when clusters associated with some of the motor tasks significantly overlapped. In the two subjects for whom merging of clusters was necessary, the technique allowed us to improve average sensitivity by more than 12% while retaining resolution of 4 tasks. In the third subject, the detection was very good even for the unmerged set, so merging did not have much effect on performance.

An advantage of the technique proposed in this paper is the considerable flexibility that it allows in choosing algorithms to be used at different levels of the classifier hierarchy. This was demonstrated by our use of a simple rule-based approach for discriminating ambulatory tasks and tasks performed in a seated position in conjunction with the use of LDA for distinguishing different types of ambulatory motor tasks. At any point of the procedure, the user may select the approach that he/she considers most likely to lead to reliable classification, and to reject merging of clusters associated with certain motor tasks because their discrimination is essential from a clinical point of view. If merging is not acceptable from a clinical point of view, the user has the option of either modifying the analysis to account for the limited sensitivity and specificity attainable with the available data set (e.g. by substituting a different classification algorithm), or adding wearable sensors to gather information that may better distinguish pairs of motor tasks associated with overlapping clusters.

It is worth mentioning that the methods demonstrated herein are not limited to the particular type of data collected in this experiment. They can be applied to nearly any time series data of one or more features derived from multiple sensors such as gyroscopes, EMG, and reconstruction of continuous kinematic variables via wearable sensors [16,41-43]. This work can also be used to identify which subsets of tasks are most difficult to identify based on the features available, and thereby help to diagnose what type of sensor would help for distinguishing the task (i.e. which type of sensor in what location would help improve the classification of motor activities).

The application of the proposed technique to data gathered from COPD patients points to an important area of research in wearable systems. Monitoring the health status of individuals undergoing cardiopulmonary rehabilitation is indeed an important clinical application of wearable systems. We believe that clinicians would be able to better manage patients with COPD if information related to the patient's level of motor activity and associated systemic responses were monitored. We also believe that monitoring would optimize exercise capacity achieved and sustained by patients with COPD after participating in a pulmonary rehabilitation program. Wearable sensors are now available to monitor respiratory rate, heart rate, and oxygen saturation in an unobtrusive way over extensive periods of time [44,45]. As wearable systems that include accelerometers and other inertial sensors have become readily available [1], the need has grown for tools such as we have proposed that facilitate the systematic design of classifiers to identify motor activities. The next step in our research on patients with COPD will be to study the association of motor activities and systemic responses. Data mining visualization techniques [46] will be key in exploring ways to present this information to clinicians in a manner suitable to prompt clinical interventions when necessary.

## Competing interests

The author(s) declare that they have no competing interests.

## Authors' contributions

All authors contributed to identifying the need for monitoring COPD patients and developing experimental procedures to gather clinically relevant data for the study. DMS and PB contributed to designing the algorithms utilized in the study. All authors contributed to the discussion of the results.
